# Clinician Perspectives of Barriers to Effective Implementation of a Rapid Response System in an Academic Health Centre: A Focus Group Study

**DOI:** 10.15171/ijhpm.2016.156

**Published:** 2017-01-01

**Authors:** John Rihari-Thomas, Michelle DiGiacomo, Jane Phillips, Phillip Newton, Patricia M. Davidson

**Affiliations:** ^1^Faculty of Health, University of Technology Sydney, Ultimo, Australia.; ^2^School of Nursing, Johns Hopkins University, Baltimore, MD, USA.

**Keywords:** Medical Emergency Team (MET), Qualitative Research, Healthcare Quality Improvement

## Abstract

**Background:** Systemic and structural issues of rapid response system (RRS) models can hinder implementation. This study sought to understand the ways in which acute care clinicians (physicians and nurses) experience and negotiate care for deteriorating patients within the RRS.

**Methods:** Physicians and nurses working within an Australian academic health centre within a jurisdictional-based model of clinical governance participated in focus group interviews. Verbatim transcripts were analysed using thematic content analysis.

**Results:** Thirty-four participants (21 physicians and 13 registered nurses [RNs]) participated in six focus groups over five weeks in 2014. Implementing the RRS in daily practice was a process of informal communication and negotiation in spite of standardised protocols. Themes highlighted several systems or organisational-level barriers to an effective RRS, including (1) responsibility is inversely proportional to clinical experience; (2) actions around system flexibility contribute to deviation from protocol; (3) misdistribution of resources leads to perceptions of inadequate staffing levels inhibiting full optimisation of the RRS; and (4) poor communication and documentation of RRS increases clinician workloads.

**Conclusion:** Implementing a RRS is complex and multifactorial, influenced by various inter- and intra-professional factors, staffing models and organisational culture. The RRS is not a static model; it is both reflexive and iterative, perpetually transforming to meet healthcare consumer and provider demands and local unit contexts and needs. Requiring more than just a strong initial implementation phase, new models of care such as a RRS demand good governance processes, ongoing support and regular evaluation and refinement. Cultural, organizational and professional factors, as well as systems-based processes, require consideration if RRSs are to achieve their intended outcomes in dynamic healthcare settings

## Background

Hospitals are facing increasing patient demand and complexity whilst also being more accountable for improving care, decreasing costs, optimising access to evidence-based treatments and minimising adverse events.^1^ An increased emphasis on clinician accountability to improve healthcare quality and safety is challenging in an environment with significant workforce shortages and variations in skill mix.^2^ As part of the global patient quality and safety agenda, the past two decades have seen a growing focus on implementing rapid response systems (RRSs) to facilitate early detection, management and escalation of deteriorating inpatients.^3^ The RRS is designed around early ‘rescue’ of patients showing abnormal physiological signs and symptoms, preventing adverse clinical events (ie, unplanned intensive care unit (ICU) admissions, unexpected cardiopulmonary arrests and/or deaths).^4,5^ Despite the progressive uptake of RRSs, various provider and systems factors have limited optimisation.^6^ The lack of translation of key principles highlights the need to consider interpersonal, intra-organisational, and systemic factors including workforce distribution, skills and shortages, culture, teamwork, power relationships, fiscal constraints, increasing public accountability^7^ and competition between discrete organisational units.^8^ Teamwork and communication are essential in ensuring patient safety.^9^ Team building is complex and influenced by professional boundaries, power relations and systems.^10^ As with any healthcare initiative, human factors and the understanding of interactions among individuals and elements of a system, may influence the level of acceptance, utilisation and ultimately, the effectiveness of RRSs within the acute care setting.^11^ The ways in which clinicians operate within the RRS depend partly on the extent to which they value its use as a tool for patient safety, as well as ways in which they engage and effectively communicate within and between professional disciplines. Despite the no blame feature of all safety and quality agendas, clinician fear of retribution often shapes reluctance to activate RRSs.^12-14^ Clarifying interactions and experiences that occur between clinicians operating within these mandated clinical systems is required to address known gaps. Frameworks in healthcare and institutional structures are still largely shaped by historical, medically dominated hierarchies^15-17^ challenging communication and innovation. Failing to acknowledge these human factors is detrimental to success when implementing any model of care.^7^

Current literature reports on barriers to effective RRS activation, including RRS knowledge, attitude of responders and workloads.^18^ This study further aimed to explore and understand how doctors and nurses experience this system and negotiate care for deteriorating patients within the RRS environment. Our objectives were to ascertain (1) factors that influence implementation and ongoing effective use of RRS and (2) clinicians’ perceptions of its efficacy and utility when the initial tier of medical response is led by the patient’s admitting team.

## Methods

The study setting was an Australian academic health centre within a jurisdictional-based model of clinical governance. The RRS had been in place for 5 years at the time the study took place and received between 250-400 activations per month. Purposive sampling was used to recruit nurse and physician participants who were employed at this site and had current knowledge of and actively participated in RRSs.^19^

A qualitative design was used to elicit perspectives of participants. We intended to facilitate discussion and narratives of experiences to understand clinicians’ meanings and motivations that informed their actions. Given the centrality of inter-professional perspectives of teams in our study, six discipline-specific and multi-disciplinary focus groups were undertaken during April and May 2014 to identify registered nurses’ (RNs) and physicians’ perceptions and experiences of the RRS.^20^ Focus groups were used to generate dynamic discussion and responses to participants’ comments, prompt memories, and refine opinions already expressed. As nurses and physicians have their own distinct cultures, histories, and approaches to teamwork, conducting several discipline-specific focus groups allowed investigation of roles and practice and for open dialogue and disclosure of potentially diverse perspectives.^15^ Owing to time constraints, some clinicians were unable to attend discipline-specific groups and chose to attend a multi-disciplinary group comprising both physicians and nurses. This choice allowed for individual narratives as well as responses and elaborative comments from others within each type of group. A literature review and preliminary discussions with key stakeholders informed development of the semi-structured topic guide (Box 1).^2,21,22^ Topics included barriers and facilitators to caring for deteriorating patients, RRS experiences, operating within and outside of the RRS protocol, and perceived need for protocol changes.

**Box 1. Semi-structured Focus Group Topic Guide**

- What factors in your ward make it easy/difficult to care for ‘sick’ patients whose condition deteriorates?
- Can you tell me how the rapid response system (RRS) works on your ward?
- What has been your experience with the RRS?
- Do you follow the Clinical Emergency Response System Protocol?
- If NO - how do you negotiate to operate outside of the Clinical Emergency Response System Protocol?
- If YES – what enables you to operate within the Clinical Emergency Response System Protocol?
- In your experience, what makes the RRS work effectively/ ineffectively?
- What, if any, changes are needed to enhance the existing RRS?

### The Rapid Response Model

Track and trigger systems are recognised both nationally and internationally as best practice models. They take many forms with triggers typically incorporating numerical (aggregate weighted) scoring, vital sign parameters or combinations of both.^23,24^ The rapid response model utilised in the study is a state-based multi-tiered vital sign parameter track and trigger system.^23^ Individual tiers are activated when a pre-determined set of clinical observation and vital sign variables are breached (track), which then ‘triggers’ the response of the appropriate level of rapid response team (RRT).^25^ The two tiers, ‘Clinical Review’ (Tier 1) consist of more sensitive trigger indicators (early warning signs), while ‘Rapid Response’ (Tier 2) contains less sensitive indicators indicative of late warning signs. Indicators are derived from research outcomes of the ‘SOCCER’ study,^26^ each attracting differing levels of clinician response (Table 1). This allows a degree of individual facility autonomy based on RRS structure, resourcing, and geographic location. Tier parameter criteria can be modified to create individual patient customisation, affectively making indicators more or less sensitive to system activation over the standardised criteria. The response processes are primarily based around initial medical response (in the Rapid Response tier) coming from admitting medical teams, or dedicated facility physicians out of normal business operating hours. Although not alone in adopting this type of response model, the majority of peer facilities more popularly initiate this level of medical response in the first (Clinical Review) tier, dispatching a critical care lead medical emergency team (MET)^13^ when Rapid Response criteria are breached.^27^ The Clinical Review tier is generally responded to and managed by unit RNs in the study facility who perform a thorough A-G (airway, breathing, circulation, disability, exposure, fluids, glucose) patient assessment within 30 minutes, initiate required interventions within their scope of practice, and escalate to the second tier if their assessment reveals possible or actual clinical deterioration. The admitting team model was chosen by this facility as it allows admitting physicians to initially manage the patient’s deterioration, thus, decreasing workload demands on individuals as the RRS response load is spread across many speciality teams, rather than just a single MET. This model was also intended to allow admitting teams opportunity to develop skills in identifying and managing clinical deterioration themselves through experience rather than relying on the MET for ‘rescue’ in every RRS situation. The admitting, or after-hours team registrar (a physician who has obtained full registration with the Medical Board of Australia with at least 3 years’ experience working in public hospital service),^28^ is required to respond to all second tier calls within 30 minutes of activation. A junior resident medical officer (physician who has obtained full registration with the Medical Board of Australia)^28^ is allocated to each clinical floor and is also required to attend. A third tier (Code Blue) is embedded within the Rapid Response tier and activates the MET from ICU if clinicians feel that immediate critical care assessment is required, there has been no physician response from a rapid response activation, or the patient is not showing sign of stabilisation or improvement 1 hour after rapid response intervention.

**Table 1:** RRS Tiers^23^

**Tier**	**Responder**	**Actions**
Clinical Review (yellow zone)	2 RNs (one of which must be considered a Senior RN) *Senior RN is subjective and not formally agreed upon in facility protocol. In the situation of responding to Clinical Review, it pertains to the nurse in charge of the unit or the most senior RN in years of experience rostered on shift	• Complete a full A-G (*airway, breathing, circulation, disability, exposure, fluids, glucose*) physical assessment on the patient within a 30-minute timeframe. • If clinically stable and not at risk of deterioration, then increase vital sign frequency and monitor for trends to unit Rapid Response (red zone) criteria. • If possible clinical deterioration or you are concerned/worried, then escalation is required to either RRS or Code Blue levels (depending on the patient’s clinical status). • Nurses are also required to perform any nursing intervention they feel is warranted within their scope of practice. If the nurse’s assessment indicates.
Rapid Response (red zone)	Patient’s admitting or primary care medical team (during business hours or Medical Registrar at all other times) plus allocated RRS Resident Medical Officer	• Complete a full A-G physical assessment on the patient within a 30-minute timeframe. • Institute therapies/interventions. • Document a medical management plan including altering any calling criteria if necessary and an escalation plan if continued/further deterioration occurs. • Escalate to Code Blue tier if: - Patient if at clinical risk of any life threatening condition and requires immediate intervention. - Allocated Medical Officer has not arrived within 30 minutes of call activation. - Patient continues to breach rapid response calling criteria 1 hour post intervention. - You are concerned/worried.
Code Blue	ICU led team of ICU/Anaesthetics Registrars and Critical Care Nurses	• Immediate response to the patient. • Institute any management required including advanced treatments and/or resuscitation.

Abbreviations: RN, registered nurse; RRS, rapid response eystem; ICU, intensive care unit.

### Recruitment

We sent invitations to attend focus groups to all nurses and physicians employed at the site via administrative email distribution lists. In addition, advertisements posted on hospital notice boards sought clinician volunteers. Although this method enabled significant reach, it precluded our ability to establish a response rate. Individuals were included if they were a nurse or physician employed at the study site and currently worked in clinical environments where the RRS operated. Interested potential participants contacted the principal researcher who provided additional oral and written study information. Recruitment ceased upon data saturation. As the principal researcher was a senior nurse within the facility and had a working relationship with many of the potential participants and a significant role within the RRS, an external experienced clinician and researcher (JLP) conducted the focus groups to minimise researcher and response bias. This individual, also a senior nurse, was neither known to participants, nor was a usual collaborator of the principal researcher, but had an understanding of and previous affiliation with the facility. Another experienced researcher moderated one group due to schedule conflict of the principal moderator; this person also performed the role of scribe in the other groups to record observational notes. Participants were informed that the principal researcher would not be attending the focus groups, but would have access to the recordings and conduct analysis. They were assured that names and identifying information would be removed from transcripts and demographic information would only be reported in aggregate form. They were also assured that the principal researcher would take steps to ensure confidentiality of participants including secure storage of data and act in accordance with established ethical frameworks. Prior to focus group commencement, all participants provided written informed consent including permission to audio record proceedings.

### Procedure

One-hour focus groups took place on weekdays at the designated health facility in a private meeting room to enable attendance of target groups. Throughout the focus groups, the moderator noted newly emerging topics and points in need of clarification that were re-visited prior to concluding the sessions along with a summary of main points. This step enabled participants to verify the moderator’s understanding and interpretation of reports, thus, acting as one method to verify findings.

### Analysis

All focus groups were audio recorded and transcribed verbatim to facilitate thematic content analysis.^29^ Analysis began with the principal researcher closely reading each transcript and listening to the audio recordings to get a sense of the proceedings and context. Transcripts were analysed using the general inductive approach.^30^ Inductive coding began with line-by-line reading and coding of raw data without a pre-specified framework to remain open to emergent topics and multiple meanings within the text. Coded text was grouped into categories of material reflecting similar topics. Categories were then synthesised into themes and independently reviewed by two additional researchers (JRT and MD). To facilitate analytical rigour, three analysts (1) principal researcher (experienced clinician perspective and context/topic expert), (2) principal moderator (experienced, yet detached clinician perspective and witness to focus group processes), and (3) external qualitative researcher (methodological expertise) posed contradictory viewpoints and new insights and contributed to consolidation of themes. This analytical triangulation facilitated capture of key aspects of the themes assessed to be most important and useful in answering the research questions.

## Results

Thirty-four health professionals (21 physicians, 13 RNs) took part in six focus groups over a five-week period (Table 2). Each group was comprised of two to five participants with the exception of the registrar group, which included 15 participants. Four groups were discipline-specific and two groups were multi-disciplinary**.** Participants included both junior and senior RNs and physicians. Participants held differing skill levels and clinical experience ranging from less than one year to greater than 10 years (Table 3)**.** Physicians had worked in both admitting specialty teams and facility-wide ‘after hours’ roles. The majority of participants were under 30 years old and had worked at the study facility for less than five years.

**Table 2:** Focus Group Composition

**Focus Group**	**Physician Participants**	**Nurse Participants**
1	0	5
2	3	0
3	0	4
4	2	2
5	2	1
6	15	0

**Table 3:** Participant Demographics^a^

	**Medical** ^b^	**Nursing** ^c^
**Years Working at the Study Facility **		
<5	21	8
>5	0	5
**Years Working in Profession ** *Participants not responded (n = 2)		
0 to 5	17	3
>5	2	10
**Type of Employment ** *Participants not responded (n = 1)		
Full time	19	11
Part time	1	2
**Age** *Participants not responded (n = 1)		
>30	14	3
>30	6	10
Male	8	0
Female	13	13

^a^ Total participants (n = 34). ^b^ Medical participants (n = 21): registrars (n = 15), residents (n = 5), and Interns (n = 1). ^c^ Nursing participants (n = 13): managers (n = 1), consultants (n = 1), educators (n=6), and registered nurses (RNs) (n = 5). *Note: Figures based on available data, some participants did not provide all demographic information requested.

Analyses of focus group data yielded a range of organisational and systems-level factors shaping the ways in which health professionals experienced and negotiated care for deteriorating patients within the RRS environment. The themes that reflect systems or organisational-level barriers to an effective RRS include (1) responsibility is inversely proportional to clinical experience; (2) actions around system flexibility contribute to deviation from protocol; (3) misdistribution of resources leads to perceptions of inadequate staffing levels inhibiting full optimisation of the RRS; and (4) poor communication and documentation of RRS increases clinician workload.

### Responsibility Is Inversely Proportional to Clinical Experience

Interns and resident medical officers (hereafter, junior physicians) reported feeling unprepared and out of their depth when they entered clinical settings. They were confused about the logistics of the RRS process, particularly around who should attend RRS calls and where (allocated areas). Despite the RRS protocol and process included as part of facility orientation, some junior physician participants remained unaware. This lack of understanding contributed to doctors developing their own unique way of making the system work and/or introducing the RRS processes from previous employment sites. This resulted in doctors frequently deviating from standardised RRS protocol and perpetuating confusion for other team members.

#### Operation of Medical Response Tier Left to Most Junior Physicians

Tier 2 of the local RRS requires senior physicians (registrar level and above) to be primary responders, with junior physicians attending as additional support and to gain learning opportunities. Junior participants reported often being first at the bedside, and on occasion, the only responder. Despite study site protocol dictating activation of a MET call in such instances, they were often unsure of their options if they were the only responder. These participants reported feeling anxious, isolated and uncertain, out of their depth, and fearful of being unsupported, as depicted in the following excerpt:

*“There are times that I felt quite out of depth….I’m still getting anxious when my pager goes off and says ‘it’s time to go and do a [rapid response] and it’s like ooooooh [nervous]”* [Junior physician].

Similarly, RN participants expressed concern around variability of junior physicians’ skill levels in managing the complexity of some deteriorating patients. Nurses’ concerns about clinical capabilities was amplified if they perceived the junior physicians as not always having the prerequisite specialist knowledge of particular medical conditions or circumstances around deterioration, considering this as possibly detrimental to patient safety:

*“…I say ‘do you know anything about VADs (ventricular assist devices)?,’ and they (junior physicians) turn around to me and say ‘no’, I’m really concerned, and I’ll guarantee the majority of the Junior Reg’s (Registrars) know nothing about VAD patients either…If you’re running a hospital with VADs and (heart) transplants and lung transplants and haematology patients you shouldn’t have a junior doctor looking after them at night, it can be quite scary”* [RN].

Amidst these circumstances, nurses often perceived that their medical colleagues were reluctant to escalate the rapid response to a higher tier when they were ‘out of their depth’ for fear of being viewed as clinically inept. Medical reluctance to seek expert support was particularly apparent at night where junior physicians feared incurring the wrath of more senior specialist staff if they perceived to have disturbed them unnecessarily.

*“…but if you push and push (for escalation) they (junior physicians) will call them (Senior Specialists) eventually because we stand our ground… but if, as you say, we have a lot of junior staff (nurses) on, and you haven’t got experienced shift leaders on, it’s very difficult to get beyond that”* [RN].

Nurses also empathised with the anxiety and complexity that physicians must face when they are required to attend a RRS call.

“*I’m sure it must be very hard for them too, going from unit to unit…If you were doing it all the time then I would have thought you would end up with good skills, but just doing it for a short period or as a fill in, it sounds as if it could be quite tricky”* [RN].

Concerns about junior nursing staff’s abilities to perform critical roles in the RRS if they lacked the experience required to distinguish important and sometimes subtle clinical cues in the first (non-medical) Tier of the RRS.

*“When you consider nurses’ experience now [new graduates], they might have six months in palliative care and six months in rehab. and suddenly they are in another [acute]unit, that’s no experience [to deal with some acute situations]. So, they don’t feel confident with their decision-making…experienced nurses have more confidence to call, a new nurse that has spent one rotation in Rehab. with knowledge of the system is one thing, but confidence in activating it is another*” [RN].

### Actions Around System Flexibility Contribute to Deviation From Protocol

There was varied understanding amongst participants around altering RRS calling criteria, enabling individual patient’s parameters to become more or less sensitive to RRS activation. Participants viewed that physicians were either inappropriately altering the criteria to prevent further RRS activation or were in contrast, reluctant to alter the criteria, thus, contributing to unnecessary and excessive activation. Nurses dissatisfied with medical responses often repeatedly activated the RRS as a way of initiating further medical review. This behaviour invariably forced physicians to alter the RSS criteria to prevent ongoing calls.

*“We keep calling a [rapid response] until it resolves, or until the criteria gets changed … keep calling it until they change it”* [RN].

Nurse participants shared their experiences in challenging physicians’ decisions to alter RRS calling criteria to unsafe levels without appropriate patient assessment, particularly for those patients already attracting multiple calls. They labelled this a ‘band-aid’ approach, omitting appropriate escalation and further investigation into why the breaches were occurring.

*“There should be a criteria if there’s multiple [RRS] calls that they are reviewed properly, not just continue on for 48 hours sitting at those [altered] levels”* [RN].

Patient safety was a concern for nurse participants, especially in relationship to junior physicians altering the RRS calling criteria. Despite protocol mandating changes can only be made by senior physicians (registrar level and above), junior physicians altered criteria at times; a strategy perceived to avoid registrar attention.

*“The only one who should change the RRS criteria is the Registrar, and that should be done in consultation with the team anyway. They [junior physicians] shouldn’t just be doing that…”* [RN].

Also concerning to nurses was the enactment of alterations by physicians who were not medically familiar with patients with complex medical care needs. In many speciality areas, nurses perceived that the RRS physicians were operating outside their area of expertise and were, therefore, not cognisant of the specific care needs of some complex specialty patients. Not having time to review the patients’ medical records before initiating changes to their treatment amplified these concerns. It was also perceived that medical records frequently lacked adequate detail, context and clarity to enable full, detailed assessments and management paths.

*“I think it’s unfair for clinicians who aren’t familiar with the patient to have to make that decision in such a short period of time, and I think it’s a lot of pressure”* [RN].

### Misdistribution of Resources Leads to Perceptions of Inadequate Staffing Levels Inhibiting Full Optimisation of the Rapid Response System

Introducing the RRS increased participant awareness of patient deterioration, but also generated a perception of further workload burden. Both nurses and physicians expressed concern that the RRS generated an increase in workloads, often without any additional resources to assist. Fewer staff working ‘after-hours’ meant that clinicians on these shifts may be less willing to enact the RRS or to deviate from established care plans.

Confusion over logistical response and over-attendance at RRS calls was reported. Such redundancies, perhaps relating to a knowledge deficit in protocol, reflect a waste of resources and frustration for some.

*“…you don’t need all of the medical staff at every [rapid response] a lot of the time the units are so busy, you spend a lot of time trying to get through them [tasks], then to put down what you are doing, then you go upstairs [to attend the call])…”* [Junior physician].

### Preference to Avoid ‘Crying Wolf’ Contributes to Complacency in Rapid Response System

A portion of RRS calls are ‘false positives’, whereby the objective criteria are breached, but the patient is not actually deteriorating. This phenomenon has contributed to a level of complacency and doubt amongst some clinicians, as depicted in the excerpts below:

*“…and you get to the next one and you think, oh I shouldn’t rush this, you know, and I think it’s a bit ‘boy who cried wolf.’ It’s sort of [rapid response] after [rapid response] where you’re not necessarily [needed] and you have another couple of flights of stairs, only to be sent away again”* [Junior physician].

### Resource Deprivation and Nurse Empathy Undermine System

Nurse and physician participants attributed strained resources to senior physicians’ inability to attend some Tier 2 RRS calls ‘after-hours.’

“*There is one [Registrar] in the whole hospital and there could be six [rapid response] calls at once, and how can they possibly get to six?...It worries me that they’re so stretched, that they can’t physically get there”* [RN].

As a result, nurses attempted to delay or avoid adding additional workload to already busy physicians.

*“Often it’s sort of ‘well we need to stop having to call the doctor,’ ‘cause the Dr looks like he’s frustrated and annoyed, and the nurse doesn’t want to call him,’ and that kind of undermines the system at times”* [RN].

This perceived pressure on human resources led to RNs feeling torn between protocol-mandated escalation and feeling responsible for creating extra workload for colleagues.

### Poor Communication and Documentation Increases Clinician Workloads

Participants suggested that inadequate communication, unclear or lack of documented nursing or medical management plans, and/or no record or clinical handover of a RRS impacted adversely on patient’s subsequent care. Several participants reported that overnight RRS events were not discussed during handover/rounds because either RRS documentation was not prominently displayed in the medical records, or senior members of the patients admitting team were not aware that a RRS call had been initiated.

*“They [night shift physicians] are generally meant to find the home teams in the morning and give them a rundown of what’s happened...if that patient has been handed-over, you should probably prioritize them first, um but I don’t see that always happening”* [Junior physician].

Participants attributed the omission of these vital details to the limited time available to physicians to convey a large volume of information.

“*So do you raise it? [the fact that the patient has had a rapid response call]* [Moderator].

*“That would be fantastic for 1 in 30 patients, but it’s hard on 4 hour rounds to keep saying ‘what about this, what about this’ when like you’re flat out ordering, doing this, doing that. It’s a gap…not much time to say what about this [rapid response] call here?”* [Junior physician].

When it worked well, clinical handover involved routine team discussions of events, including RRS activations, and involved meetings between shifts, ensuring each team member had an understanding of relevant events and plans.

“*The other really good thing is that the [rapid response] calls are handed over in the medical handover at five o’clock when we meet before the after-hours shift. That brings attention to the patients that are unwell, everyone’s got it written down on a piece of paper, everyone kind of knows a little bit about the history of the patient which makes it easier”* [Junior physician].

In some units, integrating RRS call details into unit rounds prioritised patient management for the day.

“*…When you see them on the unit round in the morning, you look at their overnight events…like it’s the first thing you do when you’re assessing your patient… it’s just your normal practice”* [Junior physician].

This variability highlights diversity in practice despite working within the same systems.

Participants discussed how inadequate technological tools, such as information management systems, were contributing factors to communication barriers and variation in handover practices. They believed that establishing better ways of identifying patients who received RRS calls or had calling criteria modified, would lead to better clinical handover and prioritisation of sicker patients on rounds. The following excerpt depicts one participant’s description of sharing information as being reliant on clinician memory and note taking in the absence of appropriate electronic tools.

*“As far as I am aware, there is no formal list or computer-based system, [rather] it’s a matter of people noting it down and taking a sticker [containing patient details] and presenting it at the handover. I think that works relatively well, it’s not very formal.”* [Junior physician].

Although described as adequate by participants, this manual system of RRS had the potential to miss identifying priority patients and those needing monitoring more closely. Completion of documentation of altered criteria was on single, loose paper forms placed in the front of patient’s bedside medical records alongside vital sign observation charts. Clinicians discussed how these forms have at times become misplaced or difficult to locate if not in the correct location every time, potentially resulting in unnecessary RRS due to poorly documented changes.

*“The altered [rapid response] calling criteria forms can get lost. If there was a better way of identifying patients who had an altered [rapid response call criteria]”* [RN].

RRS entries in the patient’s medical record were sometimes overlooked as they ‘blended’ with other entries. Participants discussed using ‘flags’ in patients’ medical records to ensure high visibility of RRS entries. The effect of poor, incomplete, misplaced or out-dated documentation around RRS deterioration and altered calling criteria disabled management plans, ultimately influencing other clinicians’ workloads. These issues created greater obstacles for after-hours RNs and physicians who sought guidance from admitting medical team documentation. Responding to after-hours RRS calls for patients who had already breached criteria during the day was reportedly frustrating for physicians when appropriate alterations had not been undertaken in a timely manner.

*“I’d just like to reiterate about getting the [admitting] team to actually make more management plans for the patient…I see the frustration on the regular night nurses’ faces because we had to [rapid response] this patient again, oh and … again, and it’s like why can’t we do something about that?...I think it would be great for the team[s] to have a very clear [documented] plan about what they want for their patients during out-of-hours”* [Junior physician].

The above excerpt reflects the need for routine review and detailed documentation of management plans. Failure to do so creates frustration and increased work for other clinicians with the potential to jeopardise patient safety. Both nurses and physicians commented on their regular workloads and responsibilities being sidelined to attend RRS calls.

*“I have had situations when working a very busy shift where you have [rapid response] calls going off …where you are supposed to attend, where you don’t get any of your work done that night, and then you hand over to the next people this huge list of which, really, you could have done because you really weren’t needed at those things [rapid response]”* [Junior physician].

The above excerpt illustrates the impact of poor documentation of patient management plans on the ability of subsequent clinicians to meet their workload demands.

## Discussion

This study highlighted multiple factors influencing clinician’s abilities to operate effectively within the RRS environment. Protocol deviation was evident to varying degrees by both disciplines, though as reported in the literature, it is not a unique observation that nurses are more likely to adhere to protocols than physicians,^31^ perhaps a manifestation of their professional training and views of role and scope of practice. This reflected consistently with nurses seemingly having greater understanding of the RRS process than their medical colleagues.

The study, however, revealed potential reasons for the occurrence of some protocol deviation. The initial information given at commencement of employment pertaining to the RRSs structure and process was less likely to be retained by physicians than nurses. Though both disciplines received identical education, senior nurses and clinical nurse educators in the clinical setting were essential in ensuring embedment of RRS knowledge and operation within the nurse culture. In contrast, an absence of ongoing support, training and evaluation of physician’s roles in the RRS was a key finding and influenced functioning within the RRS.

While a primary aim to involve and up-skill the patients’ admitting teams, barriers pertaining to the study sites’ model type were evident. Relying solely on admitting medical teams (and over-extended after-hours physicians) for primary tier medical response, at times, translated to an inconsistent and desultory RRS. Physicians, still in various stages of training, participate in these response roles for short periods, limiting both development into the role and establishing peer relationships with nurses from other clinical units. This inconsistent exposure was further complicated by a need to orientate large numbers of physicians into the responder role without support of a targeted, formal curriculum.

Existing literature discusses failures of RRS,^13,32,33^ yet studies seem scarce on examining the direct effectiveness between a variety of efferent (response) limbs models, tending to generically conclude suitability should be based on individual healthcare facilities goals and resources.^13^ While many options exist around composition and resourcing of RRTs, pros and cons are evident regardless of choice. ICU without walls^34,35^ is one concept that utilises the expertise of a trained critical care physician or team. Its small, targeted group make-up would enable easier training into rapid response roles. It would also lend to more consistent exposure to other acute areas of the hospital, theoretically supporting more effective peer relationship development outside the ICU. Similarly, MET’s and ICU Nurse Liaison models^36^ would have correlative benefits. While perhaps not encouraging the ‘enabled’ up skilling of non-critical care clinicians to the same degree as admitting team models, they do afford greater opportunity for consolidation of RRS skill and role development.

The admitting team model was not unsuccessful in identifying and managing deterioration, the study participants engaged the system, though model design did cause discord around understanding and the perceived availability, functionality and efficiency of appropriately positioned resources. It was apparent the deployment of resources used in any RRS is a major factor when determining implementation and ongoing system success. Investigation into RRS team composition and resourcing^6^ found that teams operated 24 hours a day, yet only 25% were funded, meaning resources were stripped from one area to service another. This no doubt causes extra burden on clinicians left to cover redundant positions during that time and can result in multiple forms of deviation of protocol as evidenced in this study.

There was discussion amongst participants around METs being a better option for the facility, who cited physician training, knowledge and workload as the main reasons for efficient processes. RRT makeup is still contentious within the literature with some studies showing the importance of physician inclusion,^37^ while others show beneficial results of nurse led ICU liaison/critical care outreach.^36,38^

The nursing unit team environment played an important role in support and ongoing re-enforcement of RRS utilisation. Additionally, two nurses noted the system’s ability to provide statistical evidence of workload and patient acuity. This evidence can help to highlight discrepancies between workforce supply and demand.

Physicians’ experiences reflected managing multiple competing demands, learning at various institutions with differing systems, and accelerated advancement to team member roles within the RRS. These topics were of greater concern in the junior physician groups where most agreed. Unlike nurses, these physicians do not have large support teams with senior colleague (consultant level) and educator guidance. This appears to be repeated nationally^6^ and is accentuated in situations of patient deterioration where consultant physician level guidance and support would be of most benefit. As many of these individuals are training for specialties there are anxieties about competencies and further opportunities.^39,40^ This may have lent to situations of escalation avoidance witnessed by nurses, who believed physicians needed to be seen as being able to manage and were not comfortable with patients deteriorating ‘under their watch.’

Efferent limb response demands more than just high-level skills in clinical assessment and management. Effective RRS implementation requires stronger development in responder role clarity and effective teamwork, yet there is often limited attention to this critical dynamic, both within the team and between peer relationships.^41^ The rapid response physician is required to enter unknown situations, while often unfamiliar with the patient or specialty, communicate with colleagues from different disciplines, make clinical decisions, frequently change the management of what is seen as ‘another team’s patient’ and take responsibility for the change. This responsibility imposes significant burden on physicians, many of who are relatively inexperienced. The study provides strong support for responder development of the non-technical clinical skills required to effectively perform within RRS roles; in particular, advanced communication, leadership, and teamwork being primary assets.

Future research should focus on investigating the impact and efficacy of differing RRS model types. Of particular interest, a focus on the impact of differing responders, their professional composition, level of seniority and area of origin on influencing optimal rescue of deteriorating patients. The impact each has on existing staffing and resources would also be invaluable in helping already overloaded clinicians cope with further demands of these and other imposed systems.

Ongoing development and evaluation of RRS team training is also required to ensure responding clinicians are confident and capable, not only with clinical skills, but also with ability to work in teams and effectively lead in what are, quite commonly, difficult circumstances for patients, families and fellow clinicians. Literature is still scant on the development of training specifically aimed at RRTs. Initial evidence from investigators such as Theilen et al^42^ show promising advantages in weekly multi-disciplinary simulation training, citing responder supportiveness and clinical, teamwork and communication skills as essential elements within the curricula. Large multi-centre studies to help support this evidence are required to ensure both simulation and training content are the most effective ways to train our RRTs.

Within the study site, improvements in technology are developing to aid clinicians with patient management. Electronic activation and documentation of RRS calls will prompt clinicians to better document patient clinical events and management plans while also allowing for integration of this information to other systems. Production of clinical handover alerts of these patients to proceeding shifts of clinicians for example, enables identification of patients most at risk, allowing for prioritization of rounding and closer observation. The advancement and increasing use of technologies such as these, continuous smart vital sign monitors with automated RRS activation, and technologies allowing patient bedside point of care recording, will all add to future tools for clinicians, assisting in patient deterioration prevention through swifter, more accessible and adaptable information. Add to this, increasing advancements in integrated health records allowing continuation of patient information between primary and acute health facilities.

## Limitations

Generalisability of this study is limited due to the single site. Some participant demographics are absent as a result of participants not supplying all information. The self-report and recall nature of this study is a limitation, but the qualitative approach has allowed elucidation of critical, nuanced factors influencing system implementation and ongoing optimisation.

## Conclusion

Study participants viewed the use of the RRS overall as an enabling tool for keeping patients safe, but also highlighted discrepancies and weaknesses exist in the system, particularly around choice and distribution of resourcing. The ways in which clinicians operated within this system was complex, multifactorial and non-standardised, sometimes with unintended consequences.

This study adds to an emerging body of data emphasising the importance of considering local, contextual factors, as well as model elements.^43^ Workplace processes, cultural and professional factors and systems are important considerations in implementation of RRSs. Failing to consider teamwork, communication and inter-professional dynamics impede activation of critical elements of the RRS.

## Acknowledgments

The research team acknowledge and appreciate the data collection contributions of Paula Mohacsi.

## Ethical issues

The affiliated University and Hospital Human Research Ethics Committees granted approval to undertake this study (Ethics approval number LNR/12/SVH/262).

## Competing interests

Authors declare that they have no competing interests.

## Authors’ contributions

Study conception and design (JRT, PN, PMD). Acquisition, analysis, and interpretation of data (JRT, MD, JP, PMD). Critical revision of the manuscript (JRT, MD, JP, PMD). Supervision (PMD, PN, DS).

## Authors’ affiliations

^1^Faculty of Health, University of Technology Sydney, Ultimo, Australia. ^2^School of Nursing, Johns Hopkins University, Baltimore, MD, USA.

## Key messages

**Implications for policy makers**

- Appreciate the importance of local, contextual factors, and model elements in implementing rapid response systems (RRSs).
- Organisational policy should ensure communication and negotiation via ongoing monitoring, evaluation, and coaching of health professionals.
- Ongoing training and evaluation of physicians’ roles in RRSs is critical to ensuring patient safety.
- Creation of smaller dedicated RRS teams that inhabit these roles for a longer period will enable ongoing training and support for the physician role and consolidation of skills.
- Prioritise inter-professional education and teams to increase understanding of the unique role and contribution of professional groups to the clinical encounters.

**Implications for public**

The rapid response system (RRS) concept focuses on the ‘rescue’ of patients showing abnormal signs and symptoms, preventing adverse clinical events. The way in which clinicians operate within such a system depends partly on their perception of its value as a tool for patient safety, as well as ways in which they engage and effectively communicate within and between professional disciplines. Failing to activate a RRS can risk patient safety and lead to adverse health outcomes. This study has identified an absence of ongoing training and evaluation of physicians’ roles in the RRS and the importance of teamwork and communication in ensuring patient safety.
